# Findings and Recommendations From the Joint NIST—AGA Workshop on Odor Masking

**DOI:** 10.6028/jres.116.026

**Published:** 2011-12-11

**Authors:** Nancy Rawson, Ali Quraishi, Thomas J. Bruno

**Affiliations:** AFB International, St. Charles, MO 63304; American Gas Association, Washington, DC 20001; Thermophysical Properties Division, National Institute of Standards and Technology, Boulder, CO 80305

**Keywords:** cross-adaptation, odorant conjugation, odorant masking, Zwaardemaker pair

## Abstract

Since the days of the alchemist, the observation that some substances have a smell while other substances do not has been a source of fascination. The sense of smell, or olfaction, is our least understood sense, however it is important for many human functions, including digestion, food selection and hazard avoidance. The detailed explanation of why individual chemicals (called odorants) might have a particular smell is still elusive. The situation with mixtures of odorants is even more complex and interesting. A number of distinct odorant mixture phenomena have been documented. Odorant suppression (sometimes called masking), conjugation (as described first by Zwaadermaker) and cross-adaptation are among a collection of such phenomena. They are related to the differential effects that one odorant species will have when mixed with another. Masking is a term that describes situations in which one odorant can overpower the sensation of another. There may be profound technological implications in a number of industrial sectors, most prominently in the fuel gas sector. Here, masking is suspected when the odorant that is added to natural gas can be detected by analytical instrumentation, but cannot be properly detected by an observer with a normal sense of smell. Note that this phenomenon is distinct from odor fade, which more properly describes a decrease in the concentration of an odorant rather than a decrease, disappearance or qualitative change in the perception of the odor in the absence of a change in absolute concentration. Anecdotal descriptions of masking events in the natural gas industry have persisted for over a decade, with the frequency of such events on the rise. Pursuant to the philosophy that the technological problem cannot be addressed until the basic science is understood, NIST, in collaboration with the American Gas Association (AGA), sponsored a workshop that brought together olfactory scientists and natural gas operations personnel in an effort to achieve a common understanding and identify critical research questions. This document is a summary of that workshop, and most importantly, a compendium of the findings and recommendations that resulted from the meeting.

## 1. Introduction

Olfaction, or the sense of smell, has been fully (or nearly fully) developed in vertebrates since such animals first lived on land masses. For most mammals, olfaction is a vitally important sense that is essential to survival [1]. A notable exception might be dolphins and whales, which possess no sense of smell. For humans, olfaction has become viewed as the least important and least developed sense (from a survival point of view), although its absence or loss can have serious consequences [2, 3]. For example, olfactory deficits in humans will adversely affect hazard avoidance (such as the ability to note the presence of smoke or gas leaks), food selection (and the avoidance of spoiled food) and digestion (especially the initial enzymatic and hormonal preparations for digestion).

The importance of understanding and exploiting odor is clear in the fuel gas industry [4–6]. Mixtures of mercaptans and sulfides are added to fuel gas to give the product a distinct odor that, per federal and state regulation, can be detected at 20 percent (or less) of the lower explosion limit (lel) in air [7]. This lower limit is approximately 5 % for natural gas. Thus, the odorant must enable a person with a normal sense of smell to detect the presence of natural gas at a level of 1 %. In general, federal regulations require “periodic testing”, but are not more specific as to procedure, and there appears to be significant variability over the industry with respect to the specific procedures employed to ensure regulatory compliance. Companies use a variety of standard operating procedures, odorant mixtures, and odorant injection technologies. This reflects wide differences in operational configurations, gas compositions, odorant blends and injection rates, and other factors. Most commonly, two procedures specified by ASTM D6273 are followed [8]. Moreover, numerous companies (along with their different procedures and practices) may be involved over the course of the supply chain to the end user.

The practice of natural gas odorization is in general well understood, and subject to a high level of regulation and governmental oversight [9, 10]. It is clearly a mature technology in the natural gas industry. Despite this, some problems persist. One such problem is odorant fade. Natural gas may be properly odorized at the processing station, but at some point en route to the customer, the gas may lose odorant and therefore the associated odor. There are many well understood reasons for this. These include adsorption on pipeline walls and soil [11, 12], absorption in natural gas liquids, oxidation to less odoriferous compounds, etc. [10]. When natural gas odorant fade is noted at the end of a newly-installed section of polyethylene piping, there is no real surprise, but merely an operational problem to be solved.

Not all instances of odor loss are that simple, however. A number of years ago, a utility company (local distribution company, LDC) reported that a stream of gas received from a processing plant (that used a low-temperature operation with no solvents) was being odorized with a mixture of t-butyl mercaptan and propyl mercaptan isomers. Yet, widespread testing by dozens of field personnel showed that a large fraction of this gas stream had no detectable odor. Testing by gas chromatography with sulfur chemilumenescence detection (GC-SCD) and stain tube analysis revealed the presence of the odorant fluids at appropriate concentrations. The utility had not changed any part of the pipeline operations or odorant injection. There appeared to be a problem associated with perception, not processing or gas operations. This event (and many others like it that have occurred in recent years) remains unexplained. Other events have occurred in which the gas appeared to have a very different odor than what would have been expected: instead of the usual mercaptan smell that one normally associates with natural gas, test volumes of this gas stream were reported to have a sweet or solvent-like odor.

The specific phenomenon of odor masking is a specific case of odor perception from mixtures [13–26]. Masking may include aspects of cross adaptation, suppression, conjugation, etc., and has been reviewed in a number of sources, including discussions specific to the natural gas industry [27–42]. Thus, further additional description will not be repeated here. It is clear from all recent reviews that there is an insufficient understanding of the phenomena, and this knowledge barrier prevents the rational remediation of the fuel gas problem.

## 2. Odor Masking Workshop

In April of 2010, a representative of the Thermophysical Properties Division of NIST (Bruno), and the director of Operations and Engineering of the American Gas Association (Quraishi) met with the management of the NIST Material Measurement Laboratory to discuss the phenomena associated with odor masking and what potential role NIST may play in solving this important industrial problem. Also in attendance was Mr. Robert D. Wilson (Director, Gas Materials and Standards) of National Grid. It was at this meeting that the suggestion of a workshop was presented. The goal of the workshop was to bring together representatives from the fuel gas industry and scientists who are interested in olfaction in one place to listen to one another. The philosophical basis of the workshop was that before we can address the technological problems associated with odor masking in fuel gas, we must first understand the underlying science. We note that such an overall view is shared within the gas industry [43, 44].

The two-day workshop was held at NIST-Boulder (Boulder, CO) on 3/21/11 – 3/22/11. On the basis of oral presentations, poster presentations and an open discussion forum, the desire was to develop a roadmap of necessary steps geared toward solving this problem. A summary of the presentations is provided in Table 1. The open forum session was presided over by Dr. Nancy Rawson, whose unique perspective as a research scientist and manager (her background, summarized later in this document, has included basic science, clinical studies and most recently industrial applications) made her the ideal provocateur. This document presents the conclusions (and in many cases additional questions raised) resulting from the discussions.

### 2.1 Preliminary Research Priorities

Although the technology of fuel gas odorization is mature, problems of odor fade and odor masking still occur. It can be difficult to distinguish between instances of fade and instances of possible masking. Many of these difficulties stem from a lack of basic information or data. The participants of the workshop discussed several items that might be considered peripheral to issues of masking, but which nonetheless require consideration since they are infrastructural. These are listed below as preliminary research priorities.

#### 2.1.1 Defining a Normal Sense of Smell

The requirements for natural gas odorization state that the gas must be detectable to a person with a “normal sense of smell.” We recognize that this is a less than quantitative definition that contains a great deal of ambiguity. Such definitions may be contained in prior work done at the Monell Chemical Senses Center and available in the literature, and therefore a quantitative definition in terms of concentrations may be derived from that earlier work. This body of work explicitly considered odorants such as t-butyl mercaptan, but may not have encompassed the population and conditions necessary for generation of a distribution curve that may adequately determine ‘normal’ across the population. It is well known that the sense of smell is quite variable, and is influenced by age and gender [45–48]. Even among healthy populations, true norms may require adjustment according to subgroups such as these. Accordingly, we recommend that this body of existing literature be examined specifically to determine whether it can be used to define a “normal sense of smell” for the relevant odorants. If this is not possible by use of the data already available, we recommend that a research project be initiated to address this definition.

#### 2.1.2 Vapor-Liquid Equilibrium (VLE) of Odorant Mixtures in Realistic Natural Gas

It is often useful to use thermodynamic calculations to assess or even predict whether an odor fade or odor masking circumstance might be present or imminent in a pipeline. For example, if some quantities of natural gas liquids are present, partitioning of odorant in the gas phase into the liquid phase is possible, resulting in odor fade. Unfortunately, experimental studies of VLE that explicitly consider odorant mixtures and natural gas liquids are not available. Without such measurements, thermodynamic modeling of such mixtures will be impossible. Such modeling cannot be done a priori because of the large differences in polarity and polarizability of sulfur compounds as compared to those of hydrocarbons. Moreover, since the odorant compounds are present at what could be considered trace levels, measurements will have to be extensive, especially in the infinite dilution region. We recommend the measurement and modeling of VLE of key mixtures, to enable accurate thermodynamic modeling. We note that most natural gas distribution systems are in fact dry (that is, with no liquids present), and odor masking events occur nonetheless. Thus, this research priority is viewed as necessary, but not sufficient.

Related to the explicit consideration of VLE is the consideration of solubility. There are many aspects to this, but the most important would be the solubility of odorant constituents in solvents or pseudo-solvents that might be present in natural gas lines. This may be approached on a quantitative basis by the use of thermosolvatochromatic parameters. We recommend that such solubility work be included once potential agonists are identified (see below).

#### 2.1.3 Gas Sample Integrity for Long Term Storage

Laboratories in the gas industry obtain and store samples of natural gas for analysis in a variety of containers, ranging from sampling bags to cylinders with inert coatings. It was clear from discussions at the workshop that sampling and archiving of such samples will be critical in any future study of odor masking. It is not clear, however, what the long term (12–18 month) integrity of such samples might be. Adsorption can be expected to affect the concentration of odorant species that are present at what might be considered trace quantities. A survey of experts in the field of gas metrology (from the Gas Metrology Research Group at NIST) revealed that no studies on long term storage have been done. Moreover, the opinion of these experts was that one can expect serious problems with the repeatability of chromatographic results when sulfur compounds are present [49]. We recommend a study on long term sample storage integrity, at least for the most reliable storage containers (based on inert surface gas cylinders). Concurrent with this, a standardized protocol for collection and storage of samples to be used for research purposes would be developed to ensure consistency and sample integrity across studies.

#### 2.1.4 Psychophysical Characterization of Masking Phenomena

The phenomena of a fleeting initial detection of an odor by a subject, which subsequently appears to be undetectable (‘first sniff’ effect), may be an instance of rapid adaptation or cross adaptation. This may play a role in odor masking when a subject is asked to perceive a mixture scent. It is infrastructural in that it underlies many aspects of olfaction. Anecdotally, the phenomenon may vary in relation to the odor and the age of the subject. We recommend first that an effort be made to better define the problem, perhaps defining what is meant by “first sniff effect,” which admittedly is here stated ambiguously. A better characterization is needed of the temporal characteristics of odor adaptation, including how the physical-chemical properties of the odorants, odor delivery, distribution, and diffusion plume influences those characteristics. In addition, better understanding of the masking phenomena in relation to odor detection vs. odor identification, along with consideration of the subject characteristics such as age and gender that may influence masking is needed. These data will provide the foundation for further research to generate predictive models and potential remedial actions to reduce or minimize the likelihood of masking in the field.

#### 2.1.5 Odor Clearance and Degradation

Nasal mucosa are responsible for clearing the olfactory epithelium of residual odorant, and “cleansing the palate” for subsequent olfactory experience. There is little doubt that the latency of olfaction is controlled to some extent by mucosal properties (density, and transport properties, primarily viscosity as well as composition of odorant binding and degradative proteins). Odorant removal kinetics can strongly influence both the rate of adaptation and the rate of recovery. In addition, mucosal properties vary among individuals, with age and even in response to environmental conditions such as humidity. Sorption of odorants across the epithelial sheet varies in relation to its octanol:water partition coefficient [50]. Receptors are distributed in zonal patterns across the epithelium, apparently in accordance with the deposition patterns of the odorants to which they respond [51]. Thus, altering the chemical sorptive properties of an odorant or the viscosity of the mucus could prevent the odorant from reaching the appropriate receptors, or alter its deposition pattern, which could result in altered perception. Some work has been done on the viscosity of mucus, the enzymes responsible for degradation of odorants, and the odorant binding proteins that contribute to odor delivery/removal from the receptors. However, in most cases, measurements are sparse, and these factors have not been examined in relation to the detectability of the odorants used in natural gas. Accordingly, we recommend a research program on the measurement of mucus viscosity, to establish the expected range of viscosity that might be naturally occurring, and to examine how this may impact perception of primary odorants and mixtures with varying sorption characteristics.

### 2.2 Research Priorities for Odor Masking

#### 2.2.1 Bioinformatics Study of Existing Analytical Data Sets

The natural gas industry, when faced with an occurrence of apparent masking of the odorant in natural gas, will routinely sample the gas for laboratory analysis, usually by a gas chromatographic method. The analyses vary in methodology, and there is no reason to expect consistency in terms of injection, detection, stationary phases used, peaks qualitatively identified, peaks quantitatively determined, or the calibration method employed. Indeed, there may be inconsistencies internally, within a company. Moreover, the analytical data may or may not be accompanied by a description of the problem that was being addressed. For example, did the gas have no odor, or an inappropriate (solvent-like) odor? We emphasize that this is not a reflection of poor practice, but merely recognition of the circumstances faced by those companies, coupled with a lack of standardization. Today, analyses are designed to quickly and efficiently solve the presented problem, with the best available information at the time. To the extent that analyses are available of gas samples taken during instances of apparent masking events, we recommend that these data be examined with the modern methods of bioinformatics to determine whether there is any correlation with the observation of other trace constituents. We recognize that before any such study can be completed, the data must first be evaluated for comprehensive coverage (clearly, the more trace peaks identified, the better) and relative consistency in metrology. We further recognize that the existing data that may be appropriate for such a study may be very limited.

#### 2.2.2 Analytical Protocol for Potential Masking Events

We recognize that the current analytical data available on gas samples taken during potential masking events may be very limited. We therefore recommend that going forward, a standard analytical protocol be developed and adopted for use during such events. The analyses must be extensive in that the C6+ fraction must be considered explicitly (rather than backflushed as a single peak), both qualitatively and quantitatively. Calibration must be done in a reliable and uniform way that is traceable to a consensus standard, and a statement of uncertainty will be essential for each analysis report. We anticipate that the development of this protocol will not require extensive laboratory research, but may be achieved by consensus. Some enabling research may be required, as outlined above where we considered the storage of samples, however.

#### 2.2.3 Vapor Pressure Measurement and Modeling of Potential Agonists

Since vapor pressure is one of the major enabling properties of any odorant (here, the term odorant refers to any chemical with a perceived odor), it will be important to evaluate such data for any compounds identified as potential agonists, either in the initial bioinformatics work or subsequent analytical work. The initial screening should be done with reliable databases such as the ThermoData Engine [52], the DIPPR database [53], and the NIST chemistry WebBook [54], and also accurate thermodynamic and transport property models such as REFPROP [55]. In the absence of reliable data, measurements will be required. In view of the very low volatility of the likely compounds, and the unlikely availability of highly pure samples for study, we recommend application of the concatenated gas saturation method for this work [56–58].

#### 2.2.4 Documentation of Odor Anomaly Events Going Forward

In the foregoing discussion, it is apparent that the record to date has been spotty with respect to documentation of what manifestation of an odor masking problem was noted, and who noted it. In some cases, the problem was noted by a trained operations person, in some cases by a consumer, and in some cases as the result of an accident investigation. It is typical that once a masking event has been noted, multiple testers from a distribution company would be involved in the testing. It is clear that, going forward, a uniformly more comprehensive and consistent documentation of all aspects of each potential masking event will be required. This must include the analytical measurements discussed above, as well as the detailed, written notes of all observers.

#### 2.2.5 Psychophysical Characterization of Odor Masking

There are no standardized protocols for psychophysical characterization of a 'masking' phenomenon, and different laboratories have used different approaches to examine this phenomenon. A standard documentation protocol may simply include a transcript of the incident as reported by the observer. However, quantitatively precise psychophysical methods are needed to examine those factors most salient to masking and investigate mixture properties with sufficient precision and reliability to enable the development of predictive models and test mechanistic hypotheses. Accordingly, we propose a focused research effort on the development and validation of such methods, employing odors of known relevance that are in use today as well as odors known in the fragrance industry to exert masking effects. Such studies should probably include an examination of the temporal, physical and spatial aspects of masking, and the influence of exposure parameters such as rate of dispersion or diffusion.

#### 2.2.6 Neurophysiological Basis for Odor Masking

There is ample evidence documenting the potential for odors to either suppress the intensity of, or modify the quality of other odors. The discovery of a large family of odorant receptors, the advancements in technology permitting expression of those receptors in heterologous systems and labeling specific receptors within an intact organism open new avenues of research to understand the peripheral, receptor-level events that may contribute to masking. In addition, neuroimaging and other non-invasive methods enable studies of the central events in odor perception. A key question is the extent to which masking is occurring in the periphery (e.g., antagonism at one or more specific receptor sites) or centrally (e.g., confusion at the level of signal transmission or processing). An understanding of the neurophysiological basis for masking will enable structure-function modeling for better prediction of potential maskers. However, currently most odorant receptors remain ‘orphans’ (i.e., ligands unknown), and, due to the complex pattern recognition strategy employed for encoding of odor quality by the nervous system, the notion of a receptor-based assay is likely to be problematic. Studies to examine the peripheral and central components of masking are still considered to be of utility in order to better understand how subject characteristics such as age, experience, expectation and context influence the phenomenon, and to understand and prevent the conditions under which masking is most likely to be experienced. It is recognized that certain kinds of neurophysiological studies may best be accomplished with animal models, although the degree to which these model systems may reflect human olfactory neurophysiology and perception must be considered.

#### 2.2.7 Non-Olfactory Cues and Pathways

There are several non-olfactory sensory systems that warrant consideration as the target for potential alternative/adjunct alarm agents. Such agents could serve as a backup for the odorant or could potentially enhance the sensitivity to the odorant. The trigeminal system is responsible for detection of chemical irritants such as capsaicin and menthol [59]. Trigeminal nerve endings are prevalent throughout the respiratory tract and oral cavity and control respiration and protect against inhalation of potentially toxic compounds. Interactions between irritation and olfaction have been investigated, and both suppression and enhancement may occur [60 61]. In addition, a population of sensory cells called solitary chemoreceptor cells are found in the posterior portion of the nasopharynx and throughout the respiratory system, and serve to trigger protective reflexes such as coughing and laryngeal closure [62]. These cells detect volatile and non-volatile hydrophobic molecules such as lactones [63]. Adaptation to irritant stimuli typically is far slower than adaptation to odorants, and our ability to localize the source of trigeminal stimuli is better than for odorants [45]. Sensitivity to chemical irritants also adapts, and different irritants can cross-adapt or cross-sensitize. Interestingly, our ability to localize the source of trigeminal stimuli is better than for odorants, providing a potential for additional information to be conveyed with an alarm signal [64]. At high concentrations, many odors also activate the trigeminal system, and both threshold and subthreshold and synergistic and suppressive interactions are possible [65, 66]. Agonistic sensory effects of airborne chemicals in mixtures: odor, nasal pungency, and eye irritation. Further research is needed to understand how odorant and irritant stimuli may interact with respect to detection, discrimination and adaptation properties.

## 3. Conclusions

The principal result of the NIST/AGA Odor Masking workshop was a plan of action: a delineation of the next steps required to understand the underlying science so that technological problems and effects may be addressed. The clear consensus was that before one could rationally address the engineering aspects of the various applications, the science must be less uncertain. This will not preclude intermediate results, however, and indeed we expect the olfaction research community to contribute incremental changes of practice, for example in the natural gas industry.

## Figures and Tables

**Table 1:** Listing of oral and poster presentations from the Joint NIST/AGA Workshop on Odor Masking

Oral Presentations:
*Odorization and Odor Masking in the Natural Gas Industry*, Ms. Rosemarie Halchuk, PE, Gas Quality Engineer, Xcel Energy, Denver, CO
*Chronicle of NIST Research Related to Natural Gas*, Dr. Thomas J. Bruno, Group Leader, Thermophysical Properties Division, National Institute of Standards and Technology, Boulder, CO.
*Age Associated Loss of Selectivity in Human Olfactory Sensory Neurons*, Dr. Diego Restrepo, Professor, Cell and Developmental Biology, (Director, Neuroscience Program) University of Colorado, School of Medicine, Denver, CO.
*Presence of the Solitary Chemosensory Cells in the Airways: Involvement in the response to Irritants and Neuorgenic Inflammation*, Dr. Marco Tizzano, Research Associate, Cell and Developmental Biology, University of Colorado, School of Medicine, Denver, CO
*Cognitive Influences on Adaptation and Sensitization to Odors*, Dr. Pamela Dalton, Member, Monell Chemical Senses Center, Philadelphia, PA
*Detection of Odor Mixtures by Humans*, Dr. Paul M. Wise, Member, Monell Chemical Senses Center, Philadelphia, PA
*Odor Mixture Perception in Rodents—Effects of Odor Identity, Experience, Motivation and Concentration*, Dr. Sasha Devore, Research Associate Department of Neurobiology and Behavior, Cornell University, Ithaca, NY.
*Whence Odor Masking: Olfaction as a Nonlinear System*, Dr. William Cain, Professor of Surgery (otolaryngology), School of Medicine, University of California, San Diego, CA.
*Change of Viscosity in DNA and Mucosal Systems*, Dr. Jessica Burger, Research Associate, NIST, Thermophysical Properties Division, NIST, Boulder, CO.
*Overview of Research Activities at the Rocky Mountain Taste & Smell Center*, Dr. Thomas E. Finger, Professor, Cell and Developmental Biology, University of Colorado, School of Medicine, Denver, CO.
*Overview of Research Activities at Monell Chemical Senses Center*, Dr. Pamela Dalton, Member, Monell Chemical Senses Center, Philadelphia, PA.
**Poster Presentations:**
*The American Gas Association*, Ali Quraishi, AGA.
*Thermophysical Properties Division*, Daniel G. Friend, NIST.
*Experimental Properties of Fluids Group*, Thomas J. Bruno, NIST.
*Theory and Modeling of Fluids Group*, Marcia L. Huber, NIST.
*Olfactory Oscillations and the Respiratory Cycle in Humans*, H. Gunney and T. J. C. Jacob, School of Biosciences, Cardiff University, UK.
*Methodological Factors in Odor Detection by Humans*, Toshio Miyazawa, Michelle Gallagher, George Preti, Paul M. Wise, Monell Chemical Senses Center, Philadelphia, PA.
*Why We Don’t Know More About Odor Masking and How To Solve the Problem*, William S. Cain, Chemosensory Perception Laboratory, University of California, San Diego CA.
*If You Do Not Like it Now, You Will Not Like it Later: Self Adaptation Does Not Have an Effect—Hedonic Valence of Some Odors*, Claudia Damhuis and Charles J. Wysocki, Monell Chemical Senses Center, Philadelphia, PA.
*Gas Leak Detection: Human Factors’ Considerations*, Michael S. Wogalter, North Carolina State University, Raleigh, NC, Kenneth R. Laughtery, Rice University, Houston, TX.
*Thermodynamics and Kinetics of Fluid-Clay Interactions*, Keith E. Miller and Thomas J. Bruno, National Institute of Standards and Technology, Boulder, CO.
*Heats of Adsorption and Interaction Determinations of Natural Gas Odorants on Surrogate Soil Surfaces*, Keith E. Miller and Thomas J. Bruno, National Institute of Standards and Technology Boulder, CO.
*Directed Attention Increases Sensitivity to Target But Not Background Odors*, Jeanmarie Diamond, Paul A. S. Breslin, Pamela Dalton, Monell Chemical Senses Center, Philadelphia, PA.
*Component Concentration Influences Perceptual Quality of Binary Odor Mixtures*, Ann Marie McNamara, Phillip Magidson and Christiane Linster, Cornell University, Ithaca, NY.
*Configurational and Elemental Odor Mixture Perception Can Arise From Local Inhibition*, Christiane Linster and Thom Cleland, Cornell University, Ithaca, NY.
*Intramodal Blocking Between Olfactory Stimuli in Rats*, E. L. Giannaris, T. A. Cleland and C. Linster, Cornell University, Ithaca, NY.
*An Application for a New Three Dimensional Model of the Main Olfactory Bulb in Examining the Effects of Aging on the Main Olfactory System*, Ernesto Salcedo and Diego Restrepo, University of Colorado Anschutz Medical Center.
*Viscosity of a Model Cystic Fibrosis Sputum over an Extended Temperature Range*, Jessica Burger, Arno Laesecke, Thomas J. Bruno, National Institute of Standards and Technology, Boulder, CO.
*G-protein α-Gustducin and TrpM5 Channel in Solitary Chemosensory Cells are Necessary for the Trigeminal Respiratory Depression Response Elicited by the Bitter Compound Denatonium Benzoate*, Marco Tizzano, University of Colorado, Denver, A. Vandenbeuch, University of Colorado, Denver, W. L. Silver, Wake Forest University, Winston-Salem NC, T. E. Finger, University of Colorado, Denver.
NYSEARCH *Voluntary Research, Development & Demonstration Organization and Example Commercial Leak Detector*, Daphne D’Zurko, NYSEARCH, Needham Heights, MA.
REFPROP, Eric Lemmon, Marcia L. Huber, Mark O. McLinden, National Institute of Standards and Technology, Boulder, CO.
*New Techniques for Product On Demand Design*, Michael Frenkel, Robert Chirico, Vladimir Diky, Chris Muzny, Andrei Kazakov, Joe Magee, Ken Kroenlein, Ilmutdin Abdulagatov, National Institute of Standards and Technology, Boulder, CO.
